# XV^th ^QTLMAS: simulated dataset

**DOI:** 10.1186/1753-6561-6-S2-S1

**Published:** 2012-05-21

**Authors:** Jean-Michel Elsen, Simon Tesseydre, Olivier Filangi, Pascale Le Roy, Olivier Demeure

**Affiliations:** 1 INRA UR0631 SAGA, chemin de borde rouge, BP 52627, 31326 Castanet-Tolosan, France; 2 INRA, UMR1348 PEGASE, Domaine de la Prise, 35590 Saint-Gilles, France; 3 Agrocampus Ouest, UMR1348 PEGASE, 65 rue de St Brieuc, 35042 Rennes, France

## Abstract

**Background:**

Our aim was to simulate the data for the QTLMAS2011 workshop following a pig-type family structure under an oligogenic model, each QTL being specific.

**Results:**

The population comprised 3000 individuals issued from 20 sires and 200 dams. Within each family, 10 progenies belonged to the experimental population and were assigned phenotypes and marker genotypes and 5 belonged to the selection population, only known on their marker genotypes. A total of 10,000 SNPs carried by 5 chromosomes of 1 Morgan each were simulated. Eight QTL were created (1 quadri-allelic, 2 linked in phase, 2 linked in repulsion, 1 imprinted and 2 epistatic). Random noise was added giving an heritability of 0.30. The marker density, LD and MAF were similar to real life parameters.

## Background

Statistical methods, and softwares, for the marker-assisted genetic analysis of quantitative traits and for the Genomic Evaluation of Breeding Values are partly converging in the new context of high density SNP chip technology. Genome Wide Association Studies based on independent individuals are used on a very large scale in human genetics, whereas GEBV techniques have mostly been developed for ruminant species, in particular dairy cattle where sires have very large numbers of offspring but dams only one progeny per mating. However, both GWAS and GEBV are universal approaches which should be adapted to any family structure, for instance the medium-sized full sib families found in pigs. Similarly to the 2009 and 2008 workshops [1,2], the data sets offered to exploration during the QTLMAS 2011 workshop were organized following this pig-type structure.

The architecture of analyzed traits can be highly variable. The number of QTL varies from one in the monogenic inheritance found for some disease resistances to a huge number of tiny QTLs in other cases. Moreover, the QTL may be subject to various effects including dominance, epistasis or imprinting. To appreciate the ability of methods to deal with these situations, the choice was made in our simulation to avoid polygenic noise and limit the heredity to 8 segregating QTLs, each displaying its own features.

## Simulated method

### Pedigree

The population was a collection of 20 non-independent sire families. Each sire was mated to 10 dams, a given dam being mated to only one sire. Each dam gave birth to two sets of 10 and 5 offspring, respectively. The first progeny group (n = 2000 individuals) formed the experimental population, with marker genotypes and trait phenotype information. The second group (n = 1000 individuals) were candidates to selection, only recorded for their marker information.

The parental generation (20 sires and 200 dams) was generated by a random choice of two gametes chosen in pools of 75. These 2x75 gamete pools were generated after a long history of random drift and mutation simulated by the LDSO software [3]. This history involved two steps: 1000 generations of a population comprising 1000 gametes, followed by a severe bottleneck with 150 gametes evolving during 30 generations.

### Genomes

The genome structure consisted of five autosomal chromosomes of one Morgan each. Biallelic SNPs were simulated, located every 0.05 cM (2000 SNPs /chromosome). A pool of 1000 gametes was first generated in linkage equilibrium. During the 1150 generations following this initial step, a mutation rate of 0.0002 was applied.

### Quantitative trait phenotypes

The trait variability was due to the segregation of 8 QTLs and to environmental noise. The QTLs were generated by transforming SNPs that were still polymorphic in the last generation. These SNPs were then removed from the marker data file. The QTL located on chromosome 1 was generated by pooling alleles from two adjacent SNPs, in order to create a quadri-allelic locus. QTL characteristics varied between chromosomes and were chosen to represent extreme situations (table 1). The effects of the QTLs are given in "trait units" (TU). Environmental noise variance was adjusted to the observed genetic variation, *i.e*. the genetic variation due to the additive effects of QTL, in order to give a realized heritability of 0.3. The resulting phenotypic standard deviation was 9.37 TU.

**Table 1:** Characteristics of the simulated QTLs

QTL	**Chrom**.	Position (cM)	Type	Effects			
QTL1	1	2.85	4 alleles, additive, big	Allele 1 = 0., 2 = 2., 3 = 4., 4= 6.			

					11	12	22
QTL2	2	81.9	in phase with QTL3	11	-4.	-2.	0.
QTL3		93.75	in phase with QTL2	12	-2.	0.	2.
				22	0.	2.	4.

					11	12	22
QTL4	3	5.0	opposition with QTL5	11	0.	2.	4.
QTL5		15.0	opposition with QTL4	12	-2.	0.	2.
				22	-4.	-2.	0.

				11	12	21	22
QTL6	4	32.2	Imprinted	2.0	0.0	0.0	0.0

					11	12	22
QTL7	5	36.3	epistatic with QTL8	11	2.	1.	0.
QTL8		99.2	epistatic with QTL7	12	0.	0.	0.
				22	0.	0.	0.

On chromosome 1, a QTL (QTL1) with 4 alleles, displaying large additive effects (0.0, 2.0, 4.0 and 6.0 TU for alleles 1 to 4) was positioned close to the chromosome border (2.85cM). The deviation between extreme genotypes (44 *vs*. 11) was thus 12 TU, *i.e*. about 1.28 phenotypic standard deviations. Chromosomes 2 and 3 were assigned two linked additive QTLs showing a 1-TU allelic effect, acting "in phase" on chromosome 2, and "in repulsion" on chromosome 3. The wording "phase" and "repulsion" should be clarified in our context. Four classes of chromosomes 2 (resp. 3) were observed in the last generation, defined by the alleles present at QTL2 and QTL3 (resp. QTL4 and QTL5): 1-1, 1-2, 2-1 and 2-2. The associations 1-1 and 2-2 being more frequent than the 1-2 or 2-1 in both cases, we assigned the same direction to the effects of alleles 1 (resp. 2) at QTL2 and 1 (resp. 2) at QTL3, and alleles 1 (resp. 2) at QTL4 and 2 (resp. 1) at QTL5. Chromosome 4 was characterized by an imprinted QTL of moderate effect (2 TU). All individuals receiving allele 1 from their sire displayed a quantitative phenotype increased by 2 TU as compared to individuals receiving allele 2. On chromosome 5, two epistatic QTLs were positioned far from each other. The effect of QTL7 was expressed (with mean values of 0, 1 and 2 for genotypes 11, 12 and 22) only when animals displayed genotype 11 at QTL8.

## Results

**Figure 1:**
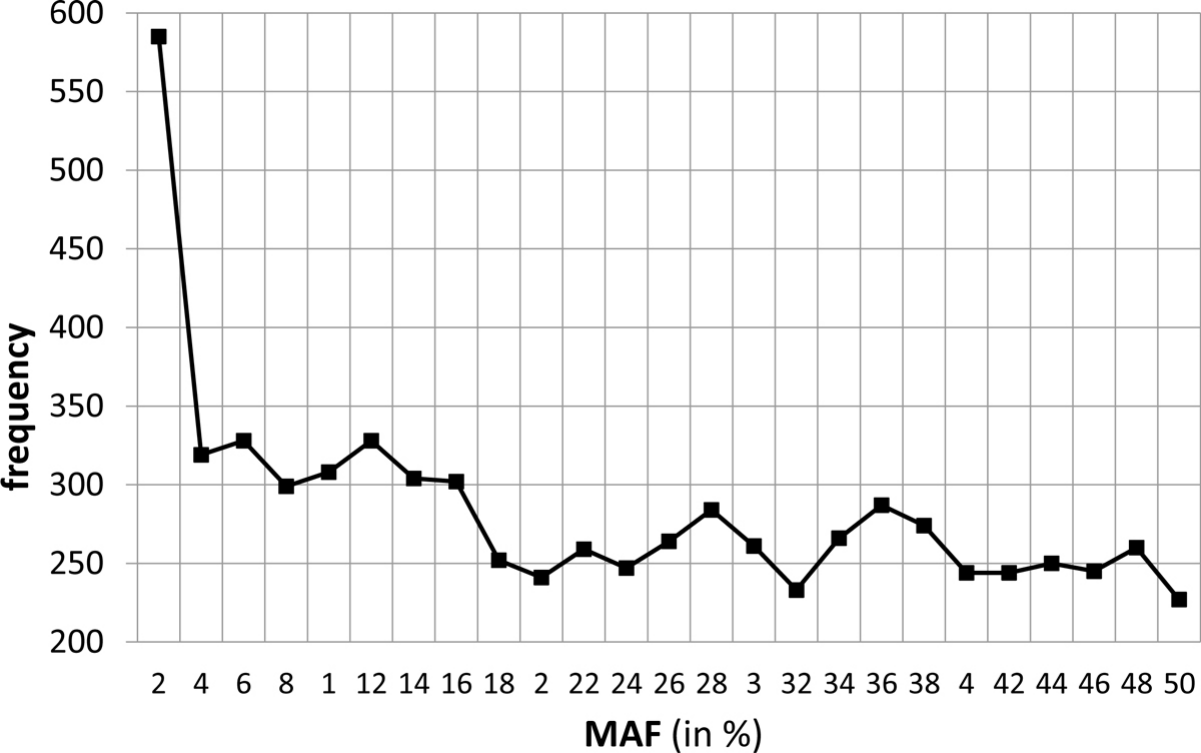
**Minor Allele Frequency distribution in the last generation**.

Amongst the 10,000 SNPs, 7,130 were still polymorphic in the last generation. The Minor Allele Frequency was classically distributed with a peak near 0 and a nearly uniform distribution elsewhere (Figure 1). The average MAF was 0.23 with a standard deviation of 0.15.

**Figure 2:**
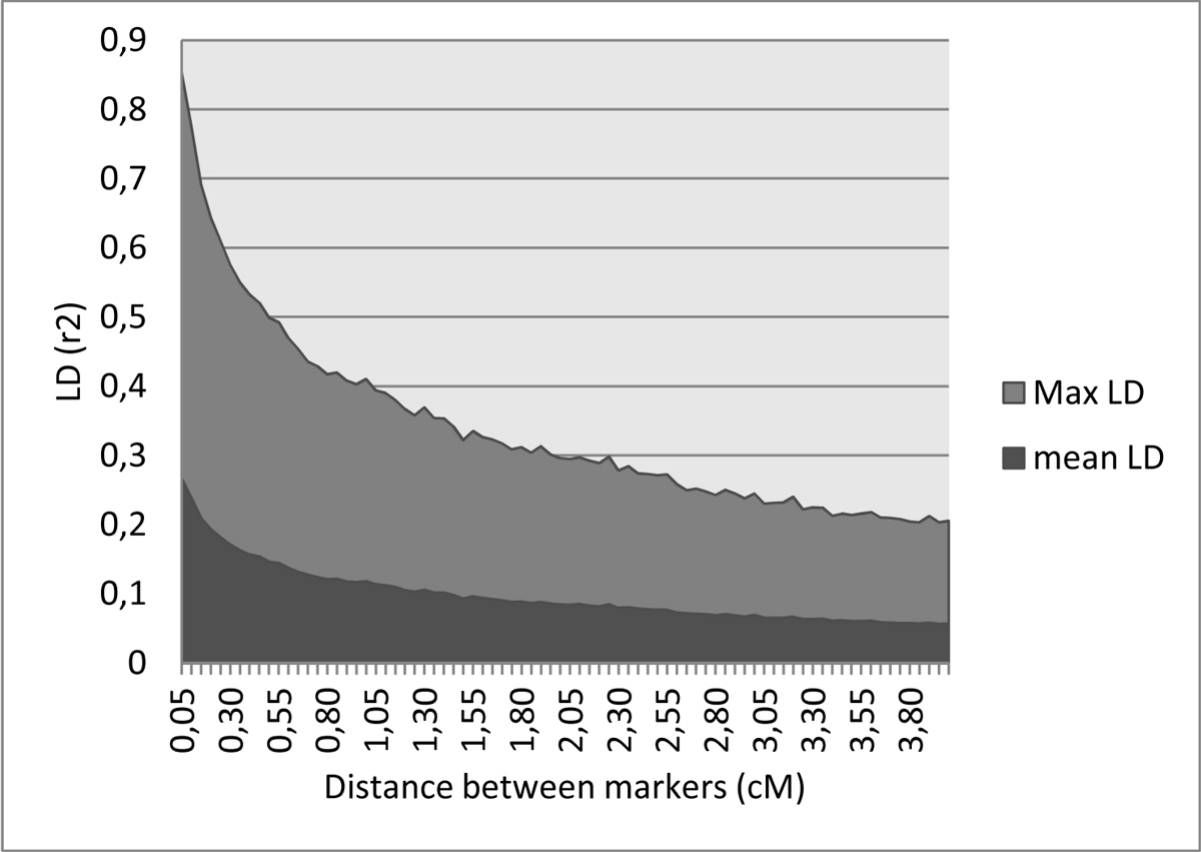
**Mean and maximum Linkage Disequilibrium (r^2^) observed in the last generation as a function of distances between markers**.

**Figure 3:**
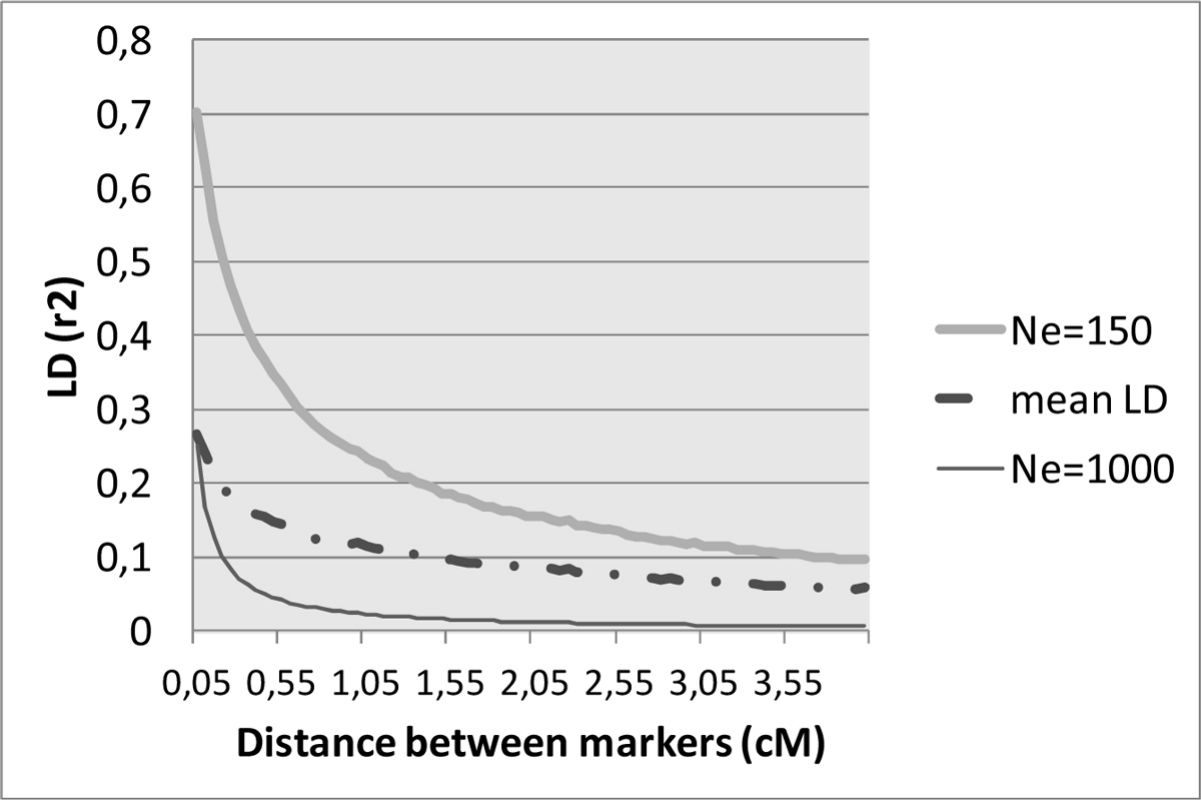
**Observed and expected (assuming effective population sizes of 150 and 1000 reproducers) Linkage Disequilibrium (r^2^) as a function of distances between markers**.

The linkage disequilibrium generated by the simulation process is typical of livestock structure (Figure 2). When compared to theoretical curves obtained using the formulae from Tenesa et al. [4], *E*(*r*^2^)=1/(4*N_e_c*+2) with *N_e _*the effective population size and *c *the recombination rate, the observed LD was closer to the *N_e_*=1000 curve at short distances, and to the *N_e_*=150 curve for larger distances between SNPs (Figure 3). This evolution is consistent with a recent bottleneck in a formerly sizeable population.

**Figure 4:**
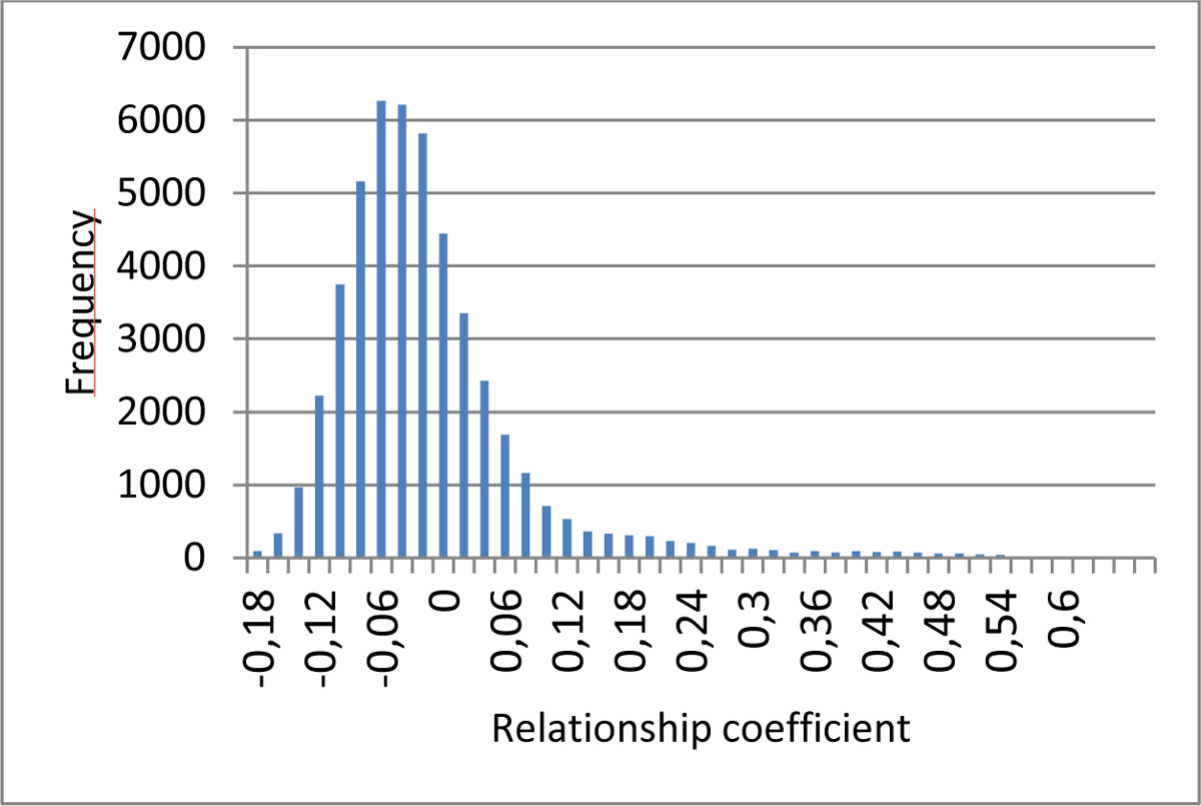
**Distribution of the genomic relationship coefficients in the parental generation**.

The 220 parents of the final generation were related, due to the limited sample size of the historical population. The distribution of the genomic relationship coefficients is given in Figure 4 as per [5]. It shows that animals were far from unrelated, a hypothesis often assumed in simple QTL detection approaches.

## Discussion

The simulated data described here were proposed to teams taking part in the QTLMAS2011 workshop in order to compare their QTL mapping and Genomic EBV techniques. The marker structure was similar to situations encountered in livestock populations, with one SNP every 0.05 cM (corresponding to a 60K SNP chip for a classical 3000 cM genome), an average MAF of 0.23, and a mean LD between close (0.05 cM) loci of 0.27, similar to findings previously described in cattle [6]. The co-ancestry relationship displayed a large variability as expected in real breeds.

On the contrary, the genetic architecture of the quantitative trait was probably much simpler than most of the situations prevailing for production traits: only 8 segregating QTLs, one or two per chromosome. Different types of allelic relationships were chosen: additivity for a single major QTL (chromosome 1), linked genes (chromosomes 2 and 3), an imprinting feature on chromosome 4 and two epistatic loci on chromosome 5. This simplified situation was chosen on purpose to avoid a possible confounding effect due to polygenic noise and to emphasize the abilities of the compared techniques to deal with such extreme cases.

## List of abbreviations used

SNP: Single Nucleotide Polymorphisms ; QTL: Quantitative Trait Locus ; MAF: Minor Allele Frequency ; LD: Linkage Disequilibrium ; GEBV: Genomic Estimated Breeding Value ; GWAS: Genome Wise Association Studies.

## Competing interests

The authors declare that they have no competing interests.

## Authors' contributions

All authors contributed to the ideas and methods, and read and approved the manuscript. ST, JME and OF programmed the simulations. JME wrote the manuscript.
